# Detectivity optimization to measure ultraweak light fluxes using an EM-CCD as binary photon counter array

**DOI:** 10.1038/s41598-021-82611-8

**Published:** 2021-02-11

**Authors:** Ibtissame Khaoua, Guillaume Graciani, Andrey Kim, François Amblard

**Affiliations:** 1 Institute for Basic Science, Center for Soft and Living Matter, Ulsan, South Korea; 2 Department of Physics, University of Connecticut, Mansfield, CT USA; 3 Department of Physics and School of Life Sciences, Ulsan National Institute of Science and Technology, Ulsan, South Korea

**Keywords:** Applied optics, Optical techniques

## Abstract

For a wide range of purposes, one faces the challenge to detect light from extremely faint and spatially extended sources. In such cases, detector noises dominate over the photon noise of the source, and quantum detectors in photon counting mode are generally the best option. Here, we combine a statistical model with an in-depth analysis of detector noises and calibration experiments, and we show that visible light can be detected with an electron-multiplying charge-coupled devices (EM-CCD) with a signal-to-noise ratio (SNR) of 3 for fluxes less than $30\,{\text{photon}}\,{\text{s}}^{ - 1} \,{\text{cm}}^{ - 2}$. For green photons, this corresponds to 12 aW ${\text{cm}}^{ - 2}$ ≈ $9{ } \times 10^{ - 11}$ lux, i.e. 15 orders of magnitude less than typical daylight. The strong nonlinearity of the SNR with the sampling time leads to a dynamic range of detection of 4 orders of magnitude. To detect possibly varying light fluxes, we operate in conditions of maximal detectivity ${\mathcal{D}}$ rather than maximal SNR. Given the quantum efficiency $QE\left( \lambda \right)$ of the detector, we find ${ \mathcal{D}} = 0.015\,{\text{photon}}^{ - 1} \,{\text{s}}^{1/2} \,{\text{cm}}$, and a non-negligible sensitivity to blackbody radiation for T > 50 °C. This work should help design highly sensitive luminescence detection methods and develop experiments to explore dynamic phenomena involving ultra-weak luminescence in biology, chemistry, and material sciences.

## Introduction

The detection of ultra-weak light sources and their possible variations with time is a challenge when investigating diverse phenomena across a wide range of fields, including weak bioluminescence, optical relaxation in various materials, delayed chemiluminescence, weak luminescence in general, or astronomy. This challenge is also a central one for the development of imaging or detection methods for biomedical applications, photo-chemical detection, and for the countless applications of luminescence across all scales. In the past 30 years, considerable efforts have been successfully devoted to best detect single photons emitted by discrete sub-wavelength size objects such as single molecules, or to localize such point-like emitters when sparsely distributed over an object field. This has led to a huge diversity of methods aimed at single photon detection and imaging^1–5^.

In many situations however, when single photons are detected, it is either impossible or physically meaningless to individually assign them to distinct "discernible" emitters. This situation prevails for instance for a liquid that contains diffusing molecules of a weakly emitting solute, or for the surface of a solid covered with a practically continuous density of weak emitters. In such cases, the physically relevant quantity to be assessed is the rate of photon emission per unit volume or unit surface of the sample. Consequently, the detection limit is set by the comparison between the rate of signal photons arriving on the whole detector area, and the rate of noise counts it generates.

In this context, the challenge of detecting very faint photon fluxes (photon s^−1^ cm^−2^) can be addressed using an electron-multiplying charge-coupled device camera (EM-CCD). Schematically, the electron-multiplication gain provides the so-called sub-electron readout noise necessary to detect single photons despite noisy output amplifiers^2,6–10^. However, electron-multiplication is a stochastic phenomenon that comes with a gain noise characterized by an excess noise factor (ENF) that typically reaches $\sqrt 2$ at high gains. This is equivalent to a 50% reduction of the effective quantum efficiency. This problem is satisfactorily solved by counting single photons using a binary discrimination. Unfortunately, binary single photon counting (PC) mode leads to coincidence losses when more than one photons arrive within the exposure time. As a consequence, the signal-to-noise ratio (SNR) exhibits a nonlinear dependence with the exposure time interval $\tau_{c}$, that results from the combination of the above binary coincidence-saturation effect with the other two main sources of noise, i.e. the clock-induced charge noise (CIC) and the dark current ($I_{d}$). The latter represents thermally induced charges that accumulate with time in each pixel in the absence of external light. The CIC is an electron transfer noise that does not depend on the pixel exposure time.

If the intensity of the source changes with time, the detection of these time-variations raises here a difficult problem. Due to detector saturation, the SNR necessarily peaks for a characteristic exposure time $\tau_{c}$, which leads to the critical sampling frequency $f_{c} = \tau_{c}^{ - 1}$ in the following sense. If the detector is operated with the exposure time $\tau = \tau_{c}$, low frequencies signal variations ($\le f_{c} /2$) are optimally detected and even oversampled, while faster variations are obviously filtered out. These higher frequencies can be captured using shorter times, but at the expense of a smaller SNR. As a consequence, if a faint flux is to be detected with no prior knowledge of it being constant or not, sampling at $\tau_{c}$ is no longer the theoretical optimum. A statistical model is then required to determine the optimal exposure time needed to best detect the variations of the signal without compromising the sensitivity to detect its time-average.

In the present work, we report on the experimental design of an EM-CCD based set-up optimized for the detection of ultra-weak light fluxes in the visible and near-infrared domain (0.4 µm ≤ λ ≤ 1 µm). An in-depth statistical analysis of the detector noises allows us to introduce a characteristic sampling time $\tau_{0}$ that maximizes the time-density of information the detector can extract from the signal. In such optimal conditions, the detectivity is maximal and the noise-equivalent-input (NEI) typically represents 2% of the dark current, i.e. 9*.*2 photon s^−1^ cm^−2^, and the photon-number detectivity amounts ${ \mathcal{D}} = 0.015\,{\text{photon}}^{ - 1} \,{\text{s}}^{1/2} \,{\text{cm}}$. We also show that fluxes can be assessed over a dynamic range of 4 decades, and we explain why this exceeds the expected range. Because of this very good detectivity, the sensitivity of the detector to blackbody radiation cannot be neglected, and we find a "noise equivalent temperature difference" (NETD) of 5 °C at 50 °C. This work should help design highly sensitive experiments to explore dynamic phenomena involving ultra-weak luminescence.

## Results

### Simple statistical model of photon counting with an EM-CCD

Let’s consider the basic operation of an EM-CCD pixel in the photon-counting mode, in which pixels receive photons from a constant light source and individually generate signal primary photon-electrons at the rate $I_{s}$ during the exposure time interval $\tau$. These photo-electrons are multiplied, shifted to an amplifier with a threshold-discrimination that generates a single bit, $0$ or $1$, that can be represented by the Bernoulli random variable $X$. In that context, the noise of a pixel refers to the mismatch between the value that bit takes, and the number of primary photo-electrons generated during $\tau$. Because of the high pixel gain, the number of electrons entering the amplifier largely exceeds the amplifier readout noise, that can therefore be safely ignored. As a consequence, the pixel noise is dominated by two contributions: the clock-induced charges (CIC) and the dark-current. Unfortunately, these contributions are also amplified by the pixel gain. The CIC corresponds to spurious electrons produced during the charge transfer and multiplication process. It can be represented by the Bernoulli random variable $X_{cic}$($= 0,1$), characterized by the probability parameter $p_{cic} = 1 - q_{cic}$ to generate an output bit $X = 1$. The parameter $p_{cic}$ does not depends on $\tau$^2,5,6^. The dark current is caused by several thermally activated processes that generate charges. It is characterized by the rate $I_{d}$ at which dark electrons are generated upstream of the multiplication process, and it can be modelled as a Poisson random variable, with parameter $\mu_{d} = I_{d} \tau$. Meanwhile, the effect of the light source can also be represented by a Poisson process, with parameter $\mu_{s} = I_{s} \tau$. Both Poisson processes add up into a single Poisson process, with parameter $\mu = \mu_{s} + \mu_{d}$. Assuming that signal photons, dark current and CIC charges are independent processes, one can simply write the probability of the output bit to be zero as $e^{ - \mu } \left( {1 - p_{cic} } \right)$. The output pixel bit $X$ is therefore characterized by the Bernoulli probability parameter $p_{X} = P\left( {X = 1} \right)$ which also sets the mean $\overline{X} = p_{X}$ and reads:

1 $$p_{X} = 1 - e^{{ - \mu_{s} }} e^{{ - \mu_{d} }} \left( {1 - p_{cic} } \right).$$

The sensitivity of the detection process therefore boils down to statistically resolve the random variable $X$ = $X_{s}$ assessed in the presence of the light source $I_{s}$, from the random variable $X$ = $X_{d}$ measured in the absence of external source in the best possible dark environment ($I_{s} = 0$). One must therefore resolve their difference $\Delta X\left( {I_{s} ,\tau } \right) = X_{s} - X_{d}$ from zero. The mean of that difference, $\overline{\Delta X} \left( {I_{s} ,\tau } \right) = p_{{X_{s} }} - p_{{X_{d} }}$, reads:

2 $$\overline{\Delta X} \left( {I_{s} ,\tau } \right) = \left[ {1 - e^{{ - \mu_{s} }} } \right]e^{{ - \mu_{d} }} q_{cic} ,$$

and can be approximated as $\overline{\Delta X} \left( {I_{s} ,\tau } \right) \approx I_{s} \tau e^{{ - I_{d} \tau }}$ in conditions far away from pixel saturation, i.e. $\mu_{s} \ll 1$. This response is linear in $I_{s}$, but not in $\tau$. Interestingly, the difference peaks for $\mu_{d} = \tau I_{d} = 1$, close to the saturation due to the dark current, and the peak value can be approximated by $I_{s}/eI_{d}$.

Regardless of any approximation, the noise that limits the detection comes from the sum $\sigma_{{X_{s} }}^{2} + \sigma_{{X_{d} }}^{2}$ of the variance of $X_{d}$ and $X_{s}$ and reads:

3 $$\sigma_{{\Delta X\left( {I_{s} ,\tau } \right)}}^{2} = p_{{X_{s} }} \left( {1 - p_{{X_{s} }} } \right) + p_{{X_{d} }} \left( {1 - p_{{X_{d} }} } \right),$$

and the signal-to-noise ratio is therefore given by:

4 $$SNR = \left( {1 - e^{{ - \mu_{s} }} } \right)q_{cic}^{ - 1/2} e^{{ - \mu_{d} /2}} /\sqrt {1 + e^{{ - \mu_{s} }} - q_{cic} e^{{ - \mu_{d} }} \left( {1 + e^{{ - 2\mu_{s} }} } \right)} .$$

For short enough exposure times compared to saturation times, i.e. $\mu_{d}$ $\ll 1$ and $\mu_{s}$ $\ll 1$, the following approximation is obtained,

5 $$SNR \approx \tau I_{s} /\sqrt {2p_{cic} + \tau \left( {2I_{d} + I_{s} } \right)} .$$

From this equation that describes the SNR before pixel saturation, we observed that the SNR increases with $\tau$, but two regimes can be distinguished, that are determined by the ratio of $2p_{cic}$ to $\tau \left( {2I_{d} + I_{s} } \right)$, i.e. by the value of $\tau$ relative to the time-scale $\tau_{c/d} = p_{cic} /I_{d}$. For very short exposure times such that $\tau \ll \tau_{c/d}$, the detector noise is dominated by the fixed contribution of the CIC, and the SNR goes linearly with $\tau$ as

6 $$SNR = \tau I_{s} /\sqrt {2p_{cic} } .$$

At longer but non-saturating times $\tau \ll \tau_{c/i}$, the dark current dominates the detector noise and the SNR goes as:

7 $$SNR = \sqrt {\tau I_{s} } /\sqrt {1 + 2I_{d} /I_{s} } .$$

At much longer times, saturation occurs due to the dark current or to the input light, depending on which one dominates, as determined by the ratio $\varepsilon = I_{s} /I_{d}$. The SNR then decreases as $e^{{ - \left({\mu_{d} + \mu_{s} }\right)/2}}$. The overall behavior of the SNR is described below from numerical simulations detailed in the next section.

To model the light detection over a large number $N_{T}$ of pixels indexed by their line and column position $\left( {ij} \right)$, we simply add the corresponding Bernoulli variables $X_{ij}$. Two random variables are constructed, $N1_{s} = \sum\nolimits_{ij} {X_{s,ij} }$ and $N1_{d} = \sum\nolimits_{ij} {X_{d,ij} }$ to represent the number of binary output counts respectively in the presence and the absence of an external light source. It is assumed that the detector receives a uniform light irradiance and has a uniform quantum efficiency over all pixels. This leads to the same Poisson parameter $\mu_{s}$ for all of the $N_{T}$ pixels collected in these two variables. For the CIC noise and the dark current, we consider that each pixel possibly has a different value $p_{cic,ij}$ and $I_{d,ij}$, to include to pixel heterogeneity in the model. Outlier pixels could indeed degrade the sensitivity, either because of their excessive dark current or CIC, and it is important to know their impact on the detector sensitivity.

As it is done above for a single pixel, we seek to resolve the random variable difference $\Delta N1\left( {I_{s} ,\tau } \right) = N1_{s} - N1_{d}$ from zero. Under the assumption that pixels are independent, their variance add up and it comes:

8 $$\sigma_{{\Delta N1\left( {I_{s} ,\tau } \right)}}^{2} = \mathop \sum \limits_{ij} p_{{X_{s} }} \left( {1 - p_{{X_{s} }} } \right) + p_{{X_{d} }} \left( {1 - p_{{X_{d} }} } \right).$$

Because of the convexity of the Bernoulli variance $p\left( {1 - p} \right)$ with $p$, the mean variance of a set of distinct Bernoulli variables with parameters $p_{ij}$ is smaller that the variance of the Bernoulli of average parameter $\left\langle {p_{ij} } \right\rangle$, and we find $\sigma_{{\Delta N1\left( {I_{s} ,\tau } \right)}}^{2} \le N_{T} \left( {\sigma_{{X_{s} }}^{2} + \sigma_{{X_{d} }}^{2} } \right)$. In other words, a lower bound for the SNR for $N_{T}$ pixels can be simply estimated from the response of a single mean equivalent pixel:

9 $$SNR \approx \sqrt {N_{T} } \left( {1 - e^{{ - \mu_{s} }} } \right)q_{cic}^{ - 1/2} e^{{ - \mu_{d} /2}} /\sqrt {1 + e^{{ - \mu_{s} }} - q_{cic} e^{{ - \mu_{d} }} \left( {1 + e^{{ - 2\mu_{s} }} } \right)} .$$

### Single-pixel characterization and heterogeneity of the EM-CCD

**Figure 1:**
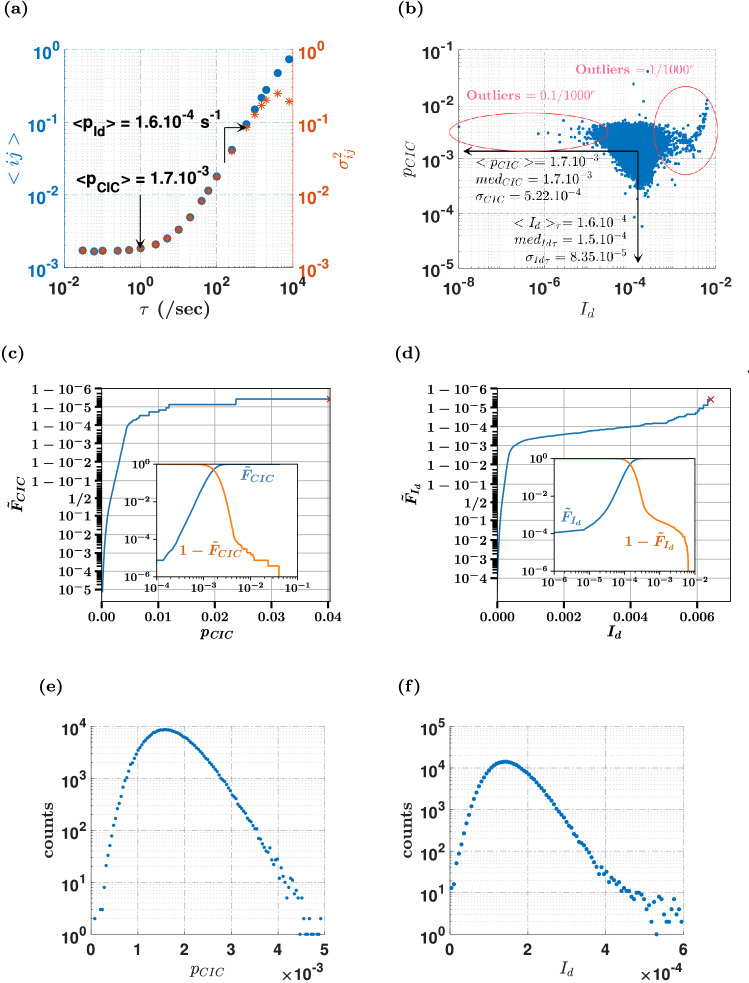
Statistics of pixel dark responses. For all pixels, the response in the binary photon counting mode was assessed from the average of the response obtained over long time series in the "complete darkness" with a cooling temperature – 85 °C, and for a range of exposure times $\tau$ (see “Materials and methods” section). The contribution of cosmic rays has been removed. (**a**) Average over all pixels of the response $< p_{X} >_{ij}$, and the variance $< \sigma_{{p_{X} }}^{2} >_{ij}$. (**b**) For each pixel, the linear fit of the dark response gives the probability $p_{CIC}$ of a CIC count, and of the probability $p_{{I_{d} }}$ of a dark current count per second. The biparametric plot represents the distribution of these parameters for each pixel, together with the statistical analysis of the two distributions. Outlier pixels, with dark current much smaller and much larger than the average represent $0.1\%$ of the pixel population each. (**c**) and (**d**) show the cumulative ($\tilde{F}_{CIC}$, $\tilde{F}_{{I_{d} }}$) and complementary cumulative ($1 - \tilde{F}_{CIC}$, $1 - \tilde{F}_{{I_{d} }}$) distribution functions of $p_{CIC}$ and $p_{{I_{d} }}$ over the detector. For inserts to (**c**) and (**d**), the vertical axes represent the logarithmic scale. For the main plots (**c**) and (**d**) instead, the vertical axes are made with a special $x \leftarrow arc{\text{tanh}}\left( {x - 1/2} \right)$ mapping, that better shows the dispersion of outliers. This analytic mapping combines a linear representation near 1/2 with a logarithmic representation of large positive or negative deviations. (**e**) and (**f**) show the 1D histograms of $p_{CIC}$ and $p_{{I_{d} }}$.

To first characterize the EM-CCD, the detector was set in the darkest possible environment (see “Materials and methods” section), and large series of images were taken over days, to sample the response for a wide range of exposure times $\tau$, with up to $10^{4}$ samples for short exposure times. For each pixel $ij$, a sample was obtained for the random variable $X_{d,ij} \left( \tau \right)$, and the sample mean was linearly fitted using Eq. (1) with $\mu_{s} = 0$. Estimates of the dark current and CIC contributions provided us with two sets of values for the whole detector, {$I_{d,ij}$} and {$p_{cic,ij}$} analyzed on Fig. 1. The average dark response over the detector, $\left\langle {p_{{X_{d,ij} }} } \right\rangle$ as a function of $\tau$, assumes the expected shape, with a lower plateau set by the CIC and a linear increases driven by the dark current, with average values of 1*.*7 × 10^−3^ and 1*.*6 × 10^−4^ s^−1^ respectively (Fig. 1a). The heterogeneity of the detector can be assessed from the joint distribution of $p_{cic,ij}$ and $I_{d,ij}$ (Fig. 1b) and the univariate statistical analyses (Fig. 1c–f). No correlation was found between these two noise characteristics, and the relative dispersion over the camera typically amount 50% for the dark count rates, and 30% for the CIC. Part of this dispersion is due to statistical outliers pixels, which typically exhibit a $10$ times excess or default of the dark count rate. These abnormal pixels represent $0.1\%$ of the detector surface, as shown by the density and the cumulative distribution functions (Fig. 1c–d).

Disregarding outliers, the bulk of the pixel heterogeneity has a clear spatial structure (Supplementary Note 1 and Supplementary Figs. 1,2), with a typical variation of $10\%$ of $p_{cic}$ between pixel columns, and a detector-wide fuzzy gradient with an excess of dark current at the top of the detector relative to its bottom. One of the basic assumption of our statistical model is that the counts generated by the CIC and dark current processes can be represented, for each pixel, as a stable Bernoulli random variable $X_{0,ij} \left( \tau \right)$. For the rest of this paper, we should question the stability of each pixel, i.e. if they behave as stationary Bernoulli processes with no correlation between successive frames, or not. This is indeed the case, has shown by the exponential distribution of the average of the time intervals between the successive counts individually delivered by each pixel (Supplementary Note 2 and Supplementary Fig. 3). This temporal stability is lost when considering all pixels, as will be observed later.

### Signal-to-noise ratio and optimal sampling time

**Figure 2:**
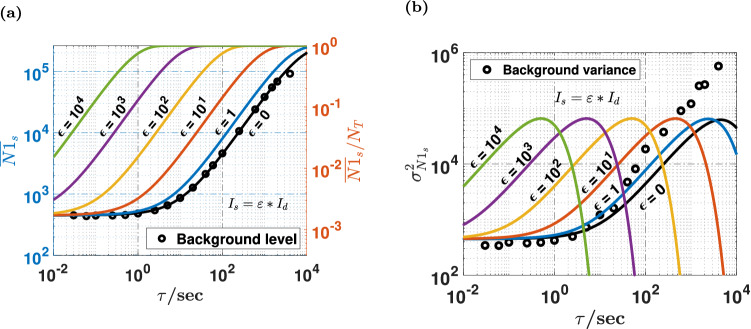
Comparison between simulation and experiments. From the CIC and $I_{d}$ values measured for each pixel, results of numerical simulation are shown (solid lines) for the averaged total detector count $\overline{N1}$ and the averaged counting probability $\overline{N1} /N_{T}$ (**a**), and for the count variance $\sigma_{N1}^{2}$ (**b**), as a function of the exposure time $\tau$ and for different values of the photon rate $\phi$ arriving on all individual pixels. $\phi$ is expressed as a fraction $\varepsilon$ of the dark current $I_{d}$. Experimental data acquired for the dark response $\phi = \varepsilon I_{d} = 0$ in the "complete darkness" are shown (black circles) after removal of cosmic rays and outlier pixels.

**Figure 3:**
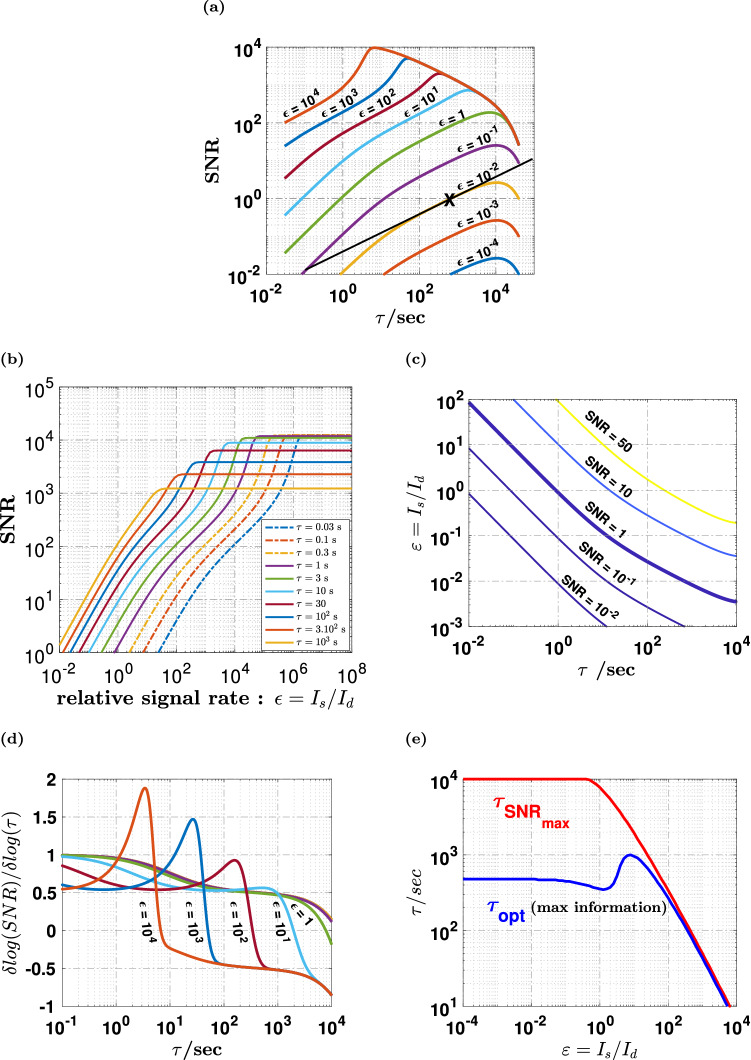
Simulation of the signal-to-noise ratio (SNR) for the whole detector. This figure shows the SNR for the whole detector as computed from the statistical model presented in the main text, using the individual pixel characteristics shown on Fig. 1a. (**a**) Shows the SNR as a function of the exposure time $\tau$ for different values of the ratio $\varepsilon = I_{s} /I_{d}$ of the signal count rate to the dark count rate. The black line with slope $1/2$, indicates the $SNR \propto \sqrt \tau$ regime discussed in the text, while the black cross indicates the value of the optimal time $\tau_{opt}$ (600 s here) described below for $\varepsilon = 10^{ - 2}$. (**b**) shows the same data as a function of $\varepsilon$ for different values of $\tau$. (**c**) Is a contour plot of the SNR. (**d**) Shows the logarithmic derivative of the SNR of plots shown in (**a**), while (**e**) shows the optimal exposure time $\tau_{opt}$ (blue line) that gives the highest density of information and therefore the best detectivity. The time for maximum SNR (red line) is much larger.

To best detect very faint light fluxes and determine the operating conditions for optimal sensitivity, our experimental knowledge of the noise parameters, {$I_{d,ij}$} and {$p_{cic,ij}$}, was combined with the SNR model described above for the whole detector, i.e. with $N_{T} = 512^{2}$ pixels. For different values of the signal rate $I_{s}$ relative to the dark count rate $I_{d}$, with the relative signal parameter $\varepsilon = I_{s} /I_{d}$, we computed numerically the mean $\overline{N1}_{s}$ and the variance $\sigma_{{N1_{s} }}^{2}$ of the total count (Fig. 2a,b), as well as the SNR (Fig. 3). In the absence of external light, i.e. when $\varepsilon = 0$, the experimental value of the average response fits well with the value of $\overline{N1}_{d}$ given by the model (Fig. 2a), but the experimental noise exceeds the prediction of the model for large light fluxes (Fig. 2b). This excess noise is probably due to the non-stationary collective behavior of the whole detector which is not seen for individual pixels, as evidenced later.

The SNR given by numerical simulations under the assumption of stationarity (Fig. 3a–c) essentially reflects how sensitively a light flux can be assessed from a single sample of the difference $\Delta N1 = N1_{s} - N1_{d}$, with and without the signal flux. As expected from the theoretical analysis, for small enough fluxes relative to the dark current, $\varepsilon < 1$, the linear regime $SNR \propto \tau$ due to the constant CIC noise is followed by the square-root regime $SNR \propto \sqrt \tau$ due to the dark current, and saturation occurs at $\tau \approx 1/I_{d}$ (Fig. 3a, Supplementary Note 3 and Supplementary Fig. 4). This 3-regimes behavior extends up to $\varepsilon \le 10$, but it gradually crosses to an unexpected behavior with a sharp SNR peak when ${\text{SNR}} > 10^{3}$. This feature observed when approaching pixel saturation can be qualitatively explained by the complex interplay of two factors. Signal saturation leads to a reduction of the effective sensitivity of the detector due to photon coincidence, but it also causes a steeper collapse the noise variance $\sigma_{{N1_{s} }}^{2} + \sigma_{{N1_{d} }}^{2}$. This effect has nothing to do with the saturation of pixels or multiplication registers per se, and this is why it leads to an increase of the SNR, with an unexpectedly large maximum ${\text{SNR}}_{max} \approx 12400$ (Supplementary Note 3 and Supplementary Fig. 4). Not surprisingly, the dynamic range is increasingly large for shorter exposure times. Such a large dynamic range is the direct consequence of operating in binary counting mode with EM gain. These simulations where executed using the complete knowledge of {$I_{d,ij}$} and {$p_{cic,ij}$}, but virtually similar results were obtained by considering an homogeneous model in which all pixel are considered to be identical to the average pixel, with $I_{d} = \left\langle {I_{d,ij} } \right\rangle$ and $p_{c} ic = \left\langle {p_{cic,ij} } \right\rangle$ (see Supplementary Note 4 and Supplementary Fig. 5).

To infer the optimal sampling time interval $\tau$, these numerical observations of the SNR can be qualitatively discussed as follows. Let’s consider two time-scales $\tau_{a}$ and $\tau_{b} = n\tau_{a} > \tau_{a}$. From the point of view of the signal, the same total photon count will be obtained, if these photons are collected as a single sample over $\tau_{b}$, or by adding $n$ adjacent samples sampled over $\tau_{a}$. If $SNR \propto \sqrt \tau$, then $\sigma^{2} \left( {\tau_{b} } \right) = n\sigma^{2} \left( {\tau_{a} } \right)$, and the variance is the same for a single sample over $\tau_{b}$ and the independent repetition of $n$ samples with time interval $\tau_{a}$. In other words, both the signal and the variance remain unchanged by the sampling fragmentation, and there is no loss of information. If $SNR \propto \tau$ instead, $\sigma^{2} \left( \tau \right)$ is a constant that does not depend on $\tau$, and the SNR associated with a single sample $\tau_{b}$ is $\sqrt n$ times larger than for the independent repetition of $n$ samples with $\tau_{a}$. Therefore, if a given exposure time $\tau$ is fragmented into a succession of multiple shorter intervals, there is no loss of information if ${\text{SNR}} \propto \sqrt \tau$, whereas repeated sampling with $n$ time-fragments degrades the SNR by $\sqrt n$ when ${\text{SNR}} \propto \tau$. The former case correspond to a $\tau$-independent detectivity, while the latter scaling leads to increased detectivity for longer sampling times.

Therefore, the sampling time should not be optimized simply by maximizing the SNR, because intensity variations faster than $\tau_{c}$ will be missed. To detect faster variations with no information loss, the best option is to choose the shortest time-scale $\tau_{opt}$ that lies within the square-root regime $SNR \propto \sqrt \tau$. This shortest time-scale is the one for which the time-density of information delivered by the detector is maximal, i.e. the one that corresponds to the highest detectivity, or the smallest noise equivalent input (NEI). The concept of NEI designates the input signal that would generate an output from a zero-noise detector, that is equivalent to the standard deviation of the noise of the actual detector injected with a zero input.

Practically, the optimal time $\tau_{opt}$ leading to maximal detectivity essentially depends on how the SNR scales with $\tau$. It is determined as the smallest value of $\tau$ for which $\partial {\text{ln}}\left( {SNR} \right)/\partial \left( {{\text{ln}}\tau } \right) = 1/2$ (Fig. 3d), and $\tau_{opt}$ could be computed as a function of the intensity of the light source (Fig. 3e). For very faint fluxes, we find that the optimal sampling time is $20$ × shorter than the time-scale of the SNR peak. Optimal sampling therefore grants access to $20$ × faster variations compared to classical sampling at $\tau_{c}$. The experimental dataset used for numerical simulations led to *τ*_*opt*_ ≈ 600 s, which gives ${\text{SNR}} = 1$ for $\varepsilon \approx 10^{ - 2}$.

### Experimental strategy and detection sensitivity

**Figure 4:**
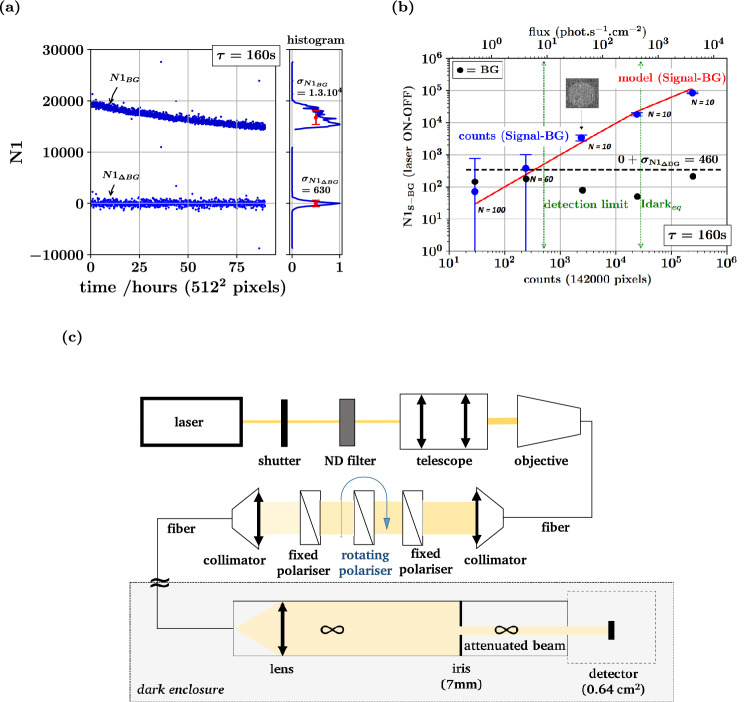
Experimental set-up and response to ultraweak calibrated fluxes. The camera cooling temperature is – 74 °C, $I_{d} = 1.13 \times 10^{ - 3}$ count/s pixel, and $p_{CIC} = 1.7 \times10^{ - 3}$. Neither cosmic rays nor outlier pixels are discarded. (**a**) Shows the count number $N1$ (top trace with blue dots) measured in complete darkness over 90 h with all pixels *τ* = 160 s. The bottom trace (blue dots) corresponds to the difference $\Delta N1$ between successive values of $N1$. Side histograms show the dispersion of both signal traces. (**b**) The difference signal $\Delta N1$ is measured with a *τ* = 160 s exposure time in the presence of a calibrated light flux injected as disk of $N_{T} = 142000$ pixels around the camera center (see inserted image). The average values $\overline{\Delta N1}$ are shown (blue dots and error bars) for 5 values of the photon flux (see “Materials and methods” section), together with the standard error of the mean. The black dots ($\bullet$) correspond to the average $\overline{\Delta N1}$ with the laser off. The black dotted line represents the detector noise, i.e. the standard deviation ($\approx 460$) extrapolated from (**a**) ($630$ for $512^{2}$ pixels) for $N_{T} = 142 \times 10^{4}$ pixels. The red line indicates the response expected from the model. The horizontal axis is marked (green arrows) with the photon flux that provides $SNR = 1$ ($9.2$ photon s$^{ - 1}$ cm$^{2}$), and also with the photon flux equivalent to the dark current ($485$ photon s$^{ - 1}$ cm$^{2}$). (**c**) Experimental setup used to measure the response to very faint fluxes. It produces a collimated beam with uniform flux on a disk smaller than the camera (see “Materials and methods” section).

The above analysis and the determination of the optimal sampling strategy implicitly involved two key assumptions, namely that each pixel behaves as stationary Bernoulli process with no internal correlation, and that there is no correlation between pixels. The obvious consequence of these assumptions is that the total noise count $N1_{d}$ assessed over a collection of $N_{T}$ pixels can also be considered as a stationary process with no internal correlation. This is not what we found when repeatedly sampling $N1_{d}$ through long time-series, for different values of $\tau$. Indeed, despite a tight temperature control within ± 0*.*02 °C, statistically significant fluctuations where observed, and the variance over the entire time-series generally exceeded the variance assessed on shorter periods of time (Supplementary Note 4 and Supplementary Figs. 6 and 7). Over very long experiments as well, a drift of the noise count was occasionally observed [50 h or more (Fig. 4a)]. In other words, the total count cannot be considered as stationary random variable, and $N1_{d}$ must be seen as a doubly stochastic random variable with a local mean $\overline{N1}_{s} \left( t \right)$ that is a random variable with idiosyncratic time-fluctuations, that are to small to be detectable from single pixel measurements. This noise excess already seen above (Fig. 2b) could arise from many causes that can hardly be mitigated beyond the proper temperature stabilization we used.

Practically, to cope with the excess noise, the signal $\Delta N1_{\tau } \left( t \right)$ was defined and assessed as the local difference between two time-adjacent samples of $N1_{s,\tau }$ and $N1_{d,\tau }$, in the presence and the absence of the signal source respectively:

10 $$\Delta N1_{s,\tau } \left( t \right) = N1_{s,\tau } \left( t \right) - N1_{d,\tau } \left( {t \pm \tau } \right).$$

In this classical scenario based on a simple adjacent background subtraction scheme, the relevant noise level is given by the dispersion $\sigma_{{\Delta N1_{d} }}$ of the difference $\Delta N1_{d,\tau } \left( t \right)$ sampled in the absence of external signal, i.e. the root mean square difference between adjacent samples of the noise count, $N1_{d,\tau } \left( t \right)$ and $N1_{d,\tau } \left( {t + \tau } \right)$. This subtraction is simply implemented by alternating samples with the camera shutter open and closed.

Experimentally, the EMCCD was operated at − 74 °C, with a measured dark current *I*_*d*_ = 1*.*1 10^−3^ s^−1^, and clock-induced charge noise of $p_{CIC} = 1.7 \times 10^{ - 3}$, leading to an optimal sampling time *τ*_*opt*_ = 160 s. While the noise count over the whole detector ($N_{T} = 512^{2}$ pixels) is clearly not stable, the adjacent background difference $\Delta N1_{d}$ exhibits a much smaller dispersion $\sigma_{{\Delta N1_{d} }} \approx 630$ (Fig. 4a). This experimental value exceeds by a factor 2 the theoretical expectation for the whole detector, $\sigma_{{\Delta N1_{d} }} = \sqrt {2I_{d} \tau_{opt} N_{T} } \approx 300$. This residual excess noise, is much less than the experimental dispersion assessed over long but unstable time-series.

To experimentally assess the sensitivity, the detector response $\Delta N1_{s,\tau } \left( t \right)$ was measured for a range of input photonic irradiance from $4$ to $4000$ photon s^−1^ cm^−2^ (Fig. 4b). This was achieved using a two-stage intensity-controlled illumination system (Fig. 4c) built to inject a uniform and monochromatic flux over a circular region of the detector (*N*_*T*_ = 142 × 10^3^ pixels and 0*.*35 cm^2^). Over this smaller number of pixels, the response to increased light fluxes $\phi_{sig}$ was found to be linear. The standard deviation for each flux matched with the expectation of a fixed noise, $\sigma_{{\Delta N1_{d} }} = 460$ obtained by extrapolating from the value 630 obtained for the whole detector. Measurement with laser light off remain below the noise dispersion limit of 460 counts. This noise regime is dominated by $\sigma_{{\Delta N1_{d} }}$, as long the shot noise of the signal can be neglected, i.e. for $N1_{s} \ll \sigma_{{\Delta N1_{d} }}^{2}$. Practically, this limit is only reached beyond saturation.

Using the noise floor $\sigma_{{\Delta N1_{d} }} = 630$ for $512^{2}$ pixels (Fig. 4a), we could express the sensitivity from the noise equivalent input flux $\phi_{nei}$ for which SNR $= 1$. With $QE = 0.9$, we obtain $\phi_{nei} = 9.2$ photon s^−1^ cm^−2^, which corresponds to $\varepsilon \approx 0.018$, i.e. 1.8% of the dark current surface density. For green photons, $\phi_{nei} = 3 10^{ - 11}$ lux, i.e. $15$ orders of magnitude less than daylight. For the sake of normalizing the observed sensitivity independently of the detector area and exposure time, we computed the detectivity and we found: ${ \mathcal{D}} = 0.015\,{\text{photon}}^{ - 1} \,{\text{s}}^{1/2} \,{\text{cm}}$.

### Thermal radiation effects and cosmic rays

**Figure 5:**
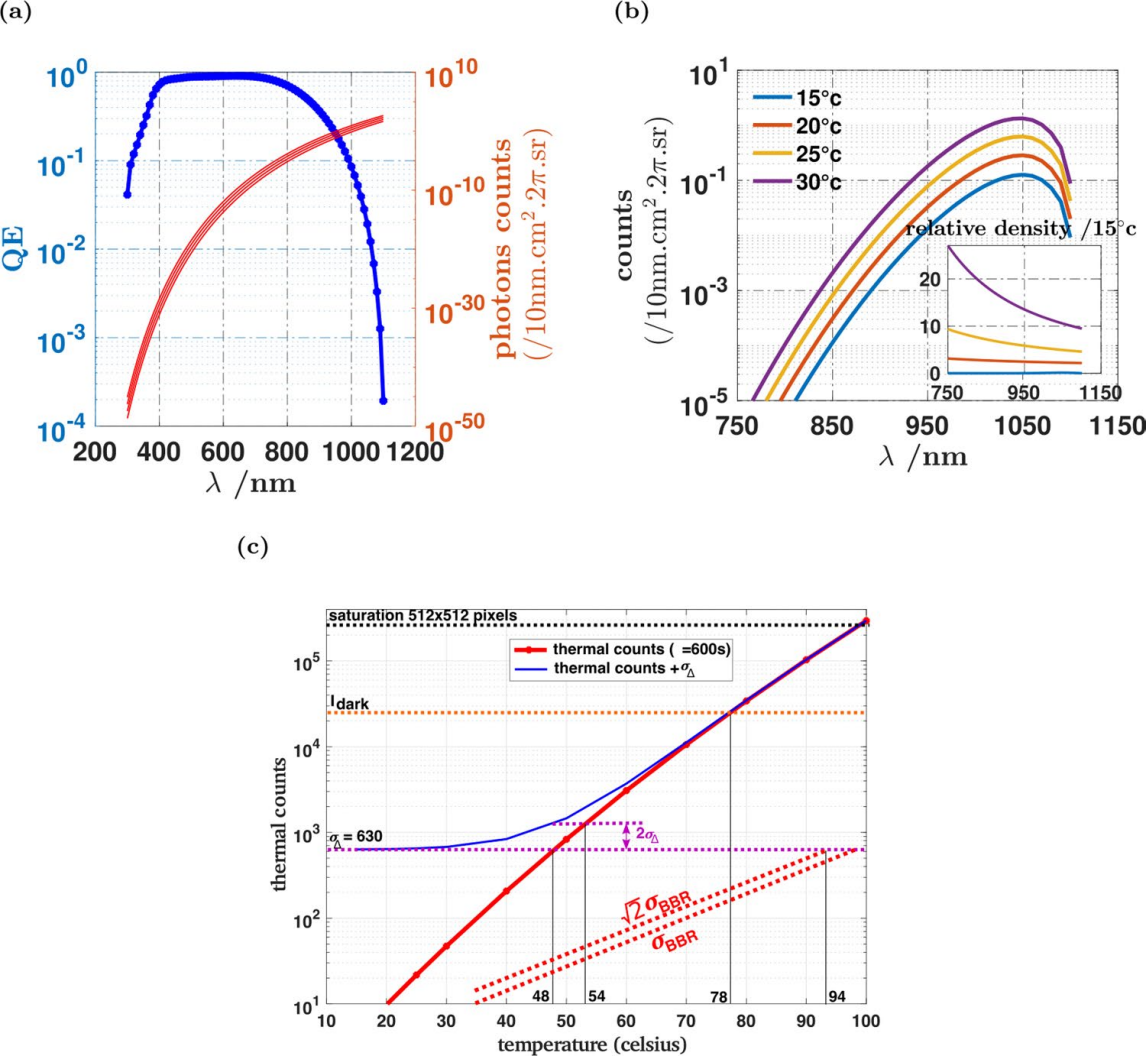
Sensitivity to thermal radiation and radiation temperature. (**a**) Shows the quantum efficiency $QE\left( \lambda \right)$ of the detector (blue line), and the blackbody radiation spectrum (red lines), taken as the photonic spectral irradiance ${\mathcal{P}}\left( {T,\lambda } \right)$ in units of number of photons per 10 nm units in the spectrum, for 4 different temperatures (15, 20, 25 and 30 °C). The spectral irradiance varies by more than 50 decades over the spectral window of the camera. The effect of temperature on the detector is computed from the effective irradiance spectrum obtained as the product $QE\left( \lambda \right){\mathcal{P}}\left( {T,\lambda } \right)$ of the spectral irradiance multiplied and the quantum efficiency. (**b**) Shows the effective thermal irradiance spectra for different temperatures, and the insert shows these spectra normalized by the 15 °C spectrum. The effective irradiance spectra of (**b**) are then integrated over the relevant part of the spectrum, over $2\pi$ steradian, and over the detector area and for *τ* = 600 s to obtain the total thermal count shown on (**c**) (red line). This count is compared to the noise floor $\sigma_{\Delta } = 630$ obtained from Fig. 4a, to the dark contribution, and to the binary saturation count $512^{2}$. The standard deviation of thermal counts is also shown (dotted red lines).

Thermal radiation remained a partial mystery until Max Planck heuristically introduced in 1905 the quantification of light-matter interactions, to explain why the blackbody radiation spectrum has a peak, with an extremely steep decrease of the radiation spectrum for wavelengths shorter than the peak wavelength^11^. Because of this so-called UV-catastrophe, usual objects at ambient temperature generate virtually no visible light, but rather emit in the infrared region with an energy peak around 10 µm. This is illustrated on Fig. 5a, which indicates factor $10^{23}$ decrease of the photonic spectral radiance between *λ* = 800 nm and 400 nm. As a consequence, thermal light is never considered when dealing with visible cameras at ambient temperature. Because of the very high detectivity obtained in our experiment, we assessed if the thermal radiation could be neglected or not.

To assess the flux of thermal photons received by the camera, we assumed that it was exposed to an ideal blackbody radiator over a $2\pi$ steradians solid angle, because the camera was practically kept in the dark and closed with dedicated blackbody materials with unity emissivity. The thermal radiation spectrum given by Planck’s Blackbody radiation law was multiplied by the quantum efficiency $QE\left( \lambda \right)$ (Fig. 5a) to obtain the spectral distribution of detected photons (Fig. 5b). Obviously, a thermal radiation peak is obtained in the near-infrared region around 1050 nm. While the position of that peak practically does not change with the radiation temperature over the range of interest, its amplitude does very much, with a $10$ fold increase between *T* = 15 °C and *T* = 30 °C. This extreme nonlinearity is in fact described by very large scaling exponents that depend on the temperature and the spectral range^12^. When integrated over the spectrum, the number of counts detected by the whole camera during *τ* = 160 s depends exponentially on the temperature (Fig. 5c) as $e^{T/7.6}$ for 20 °C < *T* < 100 °C. In this temperature range, the contribution of thermal photon counts increases from $2$ orders of magnitude smaller than the detection noise floor $\sigma_{{\Delta N1_{d} }} = 630$, to almost $3$ order of magnitude larger. Obviously, the thermal contribution exceeds the noise floor for *T* > 48 °C, further exceeds the contribution of $I_{d}$ for *T* > 78 °C, and typically saturates the binary counting mode for *T* > 100 °C. The noise floor is equivalent to shifting the radiation temperature from 48 to 54 °C, suggesting that the visible detection we use, due to its extended sensitivity in the near infra-red, must be used with care for the detection of dim light sources at temperature typically above 50 °C.

One last matter of interest here is the issue of cosmic rays, which are known to impact most light detectors and sporadically generate characteristic multipixel patterns on single frames^7^. Thanks to a simple home-made image processing routine, the impacts of cosmic rays could be removed, and we found that the number of counts they contribute typically amounts $4.2$ × $10^{ - 6}$ counts/s/pixel. This contribution corresponds to less than $3\%$ of $I_{d}$ (see Supplementary Note 5). Because of this small ratio, CR can be neglected here for most practical purposes. However, considering CR as an effective photon rate determined in binary mode is physically meaningless, because it does not reflect the actual strength of cosmic rays which generally saturate the detection process and therefore produce a much higher contribution in analog mode operation. Operating in binary mode instead represents a major advantage in that respect.

## Discussion

Using a statistical model and an experimental validation, this paper demonstrates a procedure to detect very faint light fluxes, that optimizes both the signal-to-noise ratio and the capacity to detect intensity variations. Using an EM-CCD in binary counting mode, we find that steady-state light fluxes can be detected well below the flux that corresponds to the surface density of the dark current. To measure varying light fluxes, an optimal exposure time $\tau_{opt}$ is introduced, that maximizes the time-density of the signal information and leads to a maximal detectivity. From the detection optimum, we find a detectivity that outperforms the nominal performance of most high-end EM-CCD by a factor $2 \approx 4$, as well as the best photomultiplier tubes by a similarly larger factor. As a unique consequence of binary photon counting mode, we find that the nonlinearity of the SNR with the signal leads to noise compression and enhanced dynamic range. Depending on operation condition, the dynamic range extends from 2.5 to 4 decades. Unexpectedly, our work also demonstrates that the visible camera we used is sensitive to blackbody radiation, even at moderate radiation temperatures. This work should help to design experiments aimed at exploring extremely faint luminescence across various fields.

## Materials and methods

### EM-CCD cameras

Two similar EM-CCD cameras were used, model HNü512 (Nuvu, Montreal, Canada)^8^. Their main nominal characteristics are: 512 × 512 pixels with 16 × 16 µm^2^ area, spectral range 250–1100 nm with quantum efficiency better than 90% at 600 nm and 0*.*8 ≤ *QE*(*λ*) ≤ 0*.*9 for 400 nm ≤ *λ*_*ph*_ ≤ 750 nm, back-illumination with inverted mode operation (IMO), thermo-electric cooling down to − 85 °C, EM-gain from 1 to 5000, EM register pixel capacity of 8 *× *10^8^ electrons, read-out noise with multiplication < 0*.*1 *e*^−^, clock-induced-charges < 0*.*001 e^−^/pixel/frame, dark current 0*.*0002 e^−^/pixel/s. The cameras were controlled using a homemade Python environment, using the control library provided by Nuvu. We operated these camera as follows in their binary counting mode with an EM-gain of 3000, a pixel frequency of 10 MHz, and detector cooling temperatures were either − 85 °C or − 74 °C when needed for better temperature stability.

### Dark environment and measurement of camera noises

All experiments were carried out inside a custom-made metal enclosure (0*.*8 × 0*.*8 × 0*.*8 m^3^), with inside walls painted with black anti-reflection paint. In addition, the enclosure was covered by two layers of thick black fabrics, and inside-outside ports were sealed with BK5 black fabric (Thorlabs). The same fabric was used to coat the inside walls. Specific Metal Velvet sheets with maximal absorbance and emissivity were used when needed (Aktar, Israel), for light protection purposes and as blackbody radiators. The enclosure itself was located in a customized room, for maximal light insulation. All background measurements are done in complete darkness with the camera shutter closed and sealing with BK5 blackout fabric or Metal Velvet. All materials used inside the dark enclosure were kept inside the enclosure at all times, to minimize delayed luminescence. We observed indeed that most materials do exhibit delayed luminescence, possibly with week-long decays, that became detectable in such a dark environment. Consequently, the notion of “complete darkness” was practically defined by two criteria: the background noise level could not be further reduced by additional protection from light, and the remaining variations of the background detection level were non monotonic. The temperature of the camera and the box atmosphere were constantly monitored. The former was kept stable within 0*.*05 °C and the box was kept below 25 °C at all times. Thermal control of the room led to temperature variations that were not coupled to variations of the camera temperature and the camera noises. In such conditions, the noise was characterized for each pixel, by capturing long time-series of images made in the complete darkness, with exposure times 0*.*03 s < *τ* < 9000 s. Longer series were made at short times (up to 10^4^), but shorter series over longer times. Acquisitions for this calibration procedure typically last for 2–3 days.

### Detection and removal of cosmic rays

The notion of cosmic rays (CR) entails a diversity of particles and energies, but they are defined here as the cause of randomly occurring but well defined multipixel patterns observed on single frames. In the binary counting mode, a typical pattern generally shows up as a cluster of connected pixels forming fat head connected to a decaying tail that ends up as a stretch of pixels on a single line (see Supplementary Note 5 and Supplementary Fig. 8). Because of that particular shape and topology, we could identify them with a home-made program based on detecting pixel clusters larger than a cutoff size *n*_*pix*_. In complete darkness conditions, the probability of having two counts on two adjacent pixels is very low, and the cutoff size was set such that the probability of having a cluster of size larger than *n*_*pix*_ is less than 10^−6^ in the absence of a cosmic ray event. The frequency of these events is such that they are rarely seen with exposure times typically shorter than 1 s, while up to few of them can possibly be seen for exposure times longer than 100 s. For much larger exposure times however, larger than 2000s, the spatial density of adjacent counts is too high and CR can hardly be detected by pixel connectedness. Removing CRs consisted first in identifying the pixel cluster of interest. Subsequently, the identified pixels were “replaced” by a random binary value pulled using the Bernoulli probability parameter assessed on the rest of the image.

### Experimental determination of the sensitivity

This section describes the optical set-up and operation method corresponding to Fig. 4c. To produce a very small optical power, a red laser is used (HNLS008LEC, 632*.*8 nm, 0*.*8–2 mW, polarization ratio 500:1, Thorlabs) through two successive polarization-based attenuation stages, before its injection into the dark enclosure containing the detector. The aim is to illuminate part of the camera with a uniform disk of light down to a few photons s^−1^ cm^−2^. The laser exit is first controlled by a shutter (Thorlabs SH1 and TSC00) with an Arduino board to synchronize camera acquisitions with the laser beam. A selectable set of ND absorptive filters (Thorlabs NEK 01, diameter 25 mm, 400–650 nm) is then inserted prior to a 5× beam expander (Thorlabs, GBE05-A, 400–650 nm) used to fully fill the back aperture of an objective (objective Leica 4X/0.1, Achro ∞/0.17) that injects the light in a monomode fiber (Thorlabs 630 A FC1, NA 0.10–0.14, 633–780 nm). This first fiber delivers the light through a collimator (Thorlabs, F810FC543, *f* = 34*.*74 mm, *λ* = 543 nm, NA 0.26) into the second attenuation stage designed with three identical polarizers (Thorlabs, LPVISE100A), each of which has an extinction ratio of 5000:1 at 535–690 nm. While the first and third one are parallel and fixed, the middle one is motorized (Thorlabs, KPRM1E/M, KDC101), with a computer controlled angle that sets the attenuation. The attenuated parallel beam is then focused (Thorlabs 630 A FC1, NA 0.10–0.14, 633–780 nm) into a second fiber, and the beam inside this second attenuator is protected from light using a SM1 tube (Thorlabs), between the input and the second fiber input. The light is then transported inside the dark enclosure into a sealed SM1 tube that produces the final attenuated beam and is fixed to the camera input on which it projects the desired disk shape. It contains a plano-convex lens (Thorlabs, LA1422A, *f* = 150 mm) that produces a collimated beam with a relatively uniform intensity center. That beam center is filtered by a 6*.*6 mm diameter iris diaphragm (Thorlabs, SM1D12C).

The fluctuations of the laser power are monitored by a powermeter (PM200 with S120C) using the reflection of the laser off a glass slide. The coefficient of variation is 2% (1*.*38 ± 0*.*03 mW). The output power of the second fiber was also checked by focusing the fiber output through a lens (Thorlabs, LA1951-A) onto the 3 mm diameter input of a photomultiplier (Picoquant, PMA hybrid model 40, with less than 700 dark counts/s), together with a TTL counter (Stanford Research Systems, SR620). The disk-like illumination made it possible to use part of the non-illuminated area as a reference of the background light comparatively for the laser ON and OFF and to make sure that the detected signal inside the disk does reflect the laser power. Using an exposure time *τ* = 160 s, the camera response was assessed for the following flux values (in photons/cm^2^ s): 4050, 405, 40, 4.0 and 4.8.

## Supplementary Information

Supplementary Information.

## Data Availability

The data that support the findings of this study are available from the corresponding author upon reasonable request.
