# Author Correction: Detectivity optimization to measure ultraweak light fluxes using an EM-CCD as binary photon counter array

**DOI:** 10.1038/s41598-021-90071-3

**Published:** 2021-05-19

**Authors:** Ibtissame Khaoua, Guillaume Graciani, Andrey Kim, François Amblard

**Affiliations:** 1 Institute for Basic Science, Center for Soft and Living Matter, Ulsan, South Korea; 2 Department of Physics, University of Connecticut, Mansfield, CT USA; 3 Department of Physics and School of Life Sciences, Ulsan National Institute of Science and Technology, Ulsan, South Korea

Correction to: *Scientific Reports* 10.1038/s41598-021-82611-8, published online 11 February 2021

The original version of this Article contained an error in the title of the paper, where “to measure” was incorrectly given as “to detect of”. This has now been corrected in the PDF and HTML versions of the Article, and in the accompanying Supplementary Information file.

