# Supplementary material for: Detectivity optimization to measure ultraweak light fluxes using an EM-CCD as binary photon counter array
Source: Sci Rep. 2021 Feb 11;11:3530. doi: 10.1038/s41598-021-82611-8 (PMC7878522; doi:10.1038/s41598-021-82611-8)
Supplement: Supplementary file 1 — Supplementary Information. [file 41598_2021_82611_MOESM1_ESM.pdf]

Detectivity optimization to measure ultraweak light fluxes using an EM-CCD as binary photon counter array

----

Supplementary notes and supplementary figures

Ibtissame Khaoua, Guillaume Graciani, Andrey Kim and François Amblard

Supplementary note 1 :
Spatial pattern of the camera noise parameters p_{CIC} and I_d.

This note makes use of the detail analysis of the characteristics (p_{CIC} and I_d) of all individual pixel (see main figure 1). In this note, the data is mapped back to the geometry of the detector, i.e. its 512 lines and 512 columns.

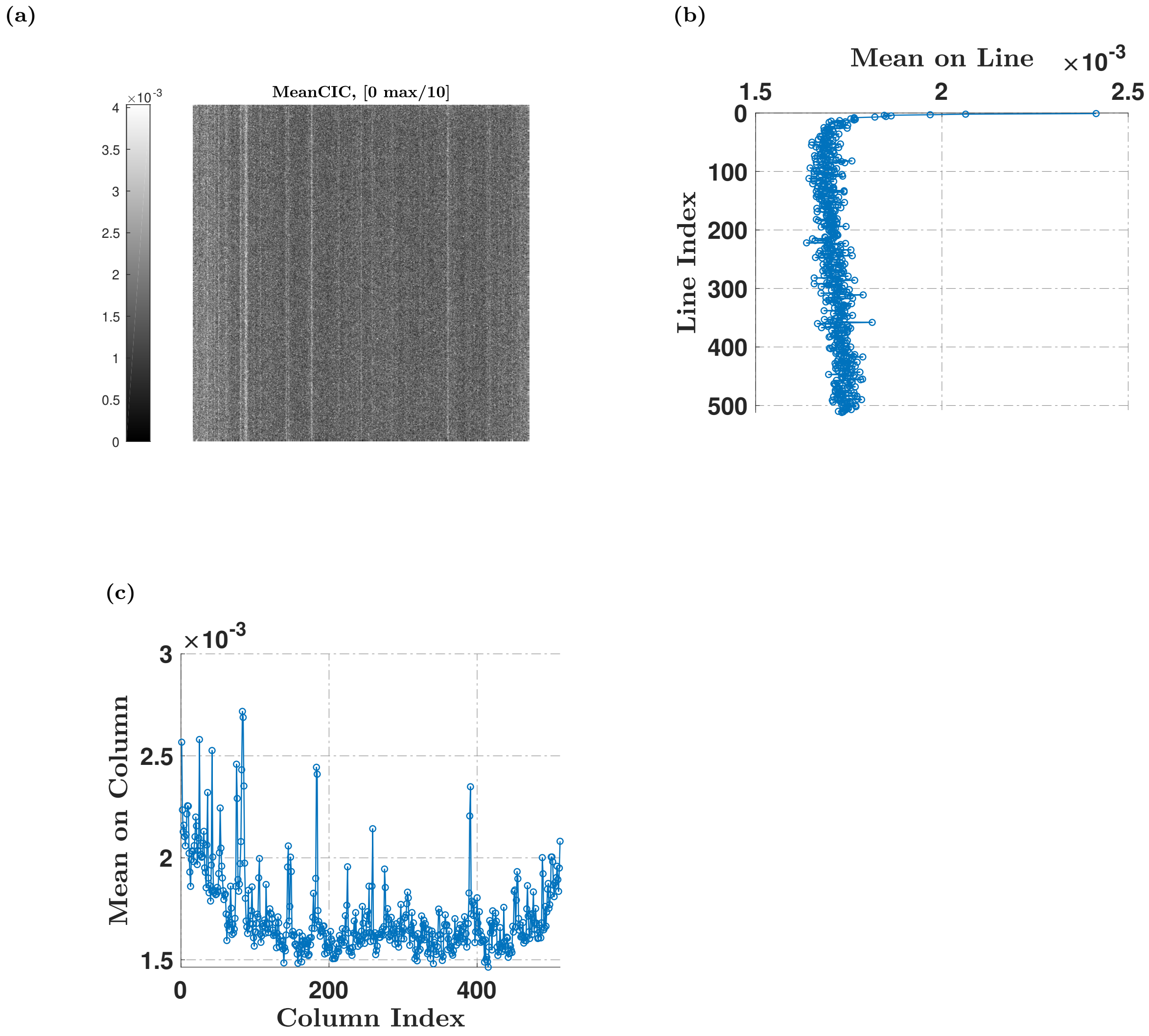

Supplementary figure 1 – **Spatial Pattern of the Clock Induced Charge probability** (p_{CIC}). The probability of p_{CIC} was assessed experimentally for all pixels (see main figure 1). The distribution is shown here (a) as a greyscale image. Because outlier values largely exceeds the standard deviation of the bulk distribution, the greyscale maximum was set to $1/10th$ of the maximum of the distribution, thus leaving a few outliers out of this representation. Distribution of the average of p_{CIC} over each line (c). Distribution of the average of p_{CIC} over each column (b).

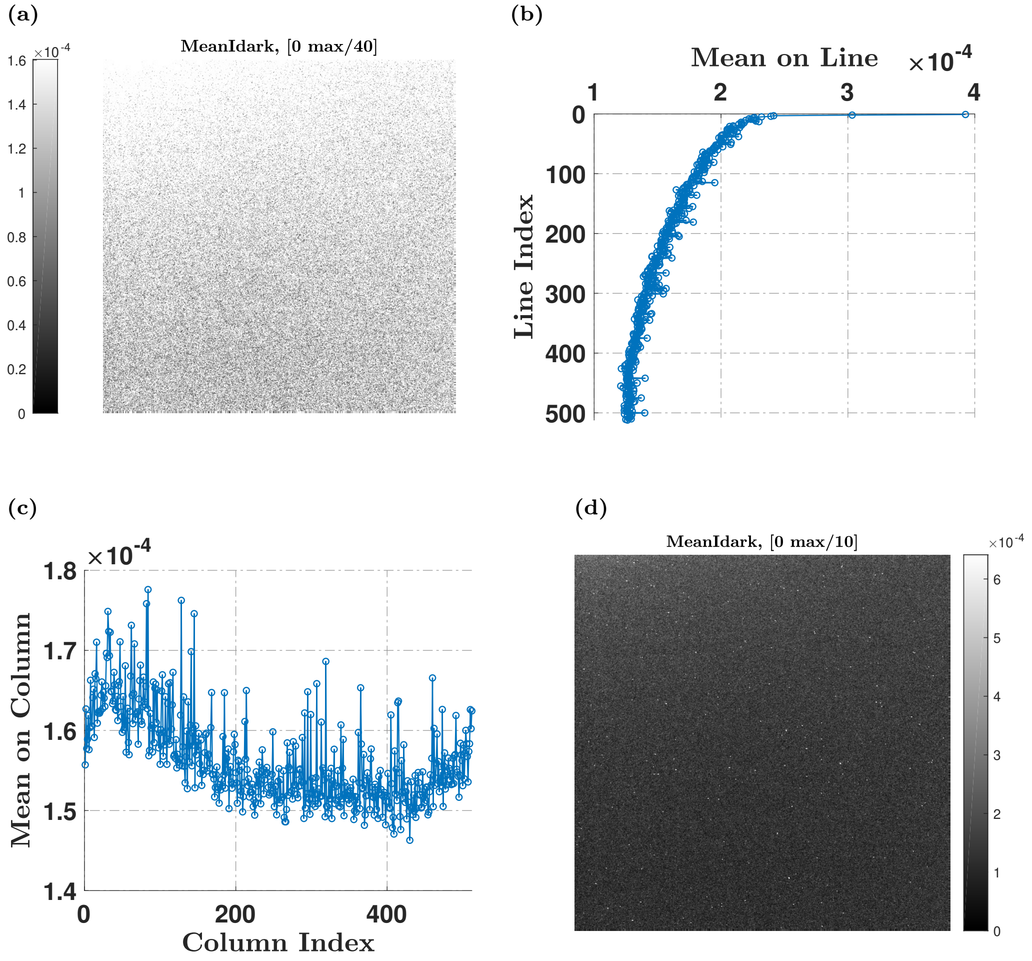

Supplementary figure 2 – **Spatial Pattern of the dark current** (I_d). The dark current was assessed experimentally for all pixels (see main figure 1). The distribution is shown here (a) as a greyscale image. Because outlier values largely exceeds the standard deviation of the bulk distribution, the greyscale maximum was set to $1/40th$ of the maximum of the distribution, thus leaving a few outliers out of this representation. Distribution of the average of I_d over each line (b). Distribution of the average of p_{CIC} over each column (c). Image with greyscale maximum was set to $1/10th$ of the maximum of the distribution (d).

Supplementary note 2 : Poissonness of individual pixels.

Our model assumes that each pixel behaves as a stationary Bernouilli random variable X. One consequence of this assumption is that the complementary cumulative distribution fonction of the time intervals between successive time-points where $X = 1$ is expected, should assume an negative exponential dependance on τ. We tested this prediction, and the result is shown here.

(a)

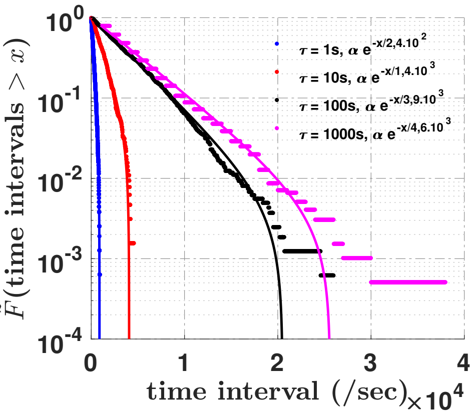

Supplementary figure 3 – **Distribution of time intervals.** For each pixel, we measured the complementary cumulative distribution fonction of the time intervals between successive time-points where $X = 1$. The average over all pixels of the individual complementary cumulative distribution fonctions is shown, together with the fit with the expected exponential form. The average function is shown for different values of the exposure time τ.

Supplementary note 3:
Further analyses of the SNR and pixel heterogeneity.

It is the purpose of this note to present a more extensive analysis of the SNR, and to discuss the impact of pixel heterogeneity.

Supplementary figure 4 shows the SNR as a function of the exposure time τ and the relative intensity of the signal $\epsilon = I_s/I_d$, using a wide range of parameter values, with $10^{-4} < \epsilon < 10^8$ and $30\,\text{ms} < \tau < 10^4\text{s}$.

Pixel heterogeneity comes with a spatial pattern (Supplementary note 1) for the bulk of the pixel distribution, together with 2% pixel population of randomly distributed outliers. The knowledge of the spatial heterogeneity is usefull the camera is used is used in analog mode for imaging purpose. But the question stands out if it matters in binary mode for non-imaging purposes. Unlike average pixel count $\overline{N1}$ of $N1$ which obviously is not impacted by the heterogeneity, the noise entering the SNR does. To assess how much the SNR depends on pixel heterogeneity, it was numerically computed using the same data as used for the main figures 1-2-3. In main figure 3, individualy pixel values were considered (heterogeneous model). Here, the SNR was computed using the same heterogeneous data, but also by considering that all pixels have the same value for p_{CIC} and I_d (homogeneous model). Supplementary figure 5 shows that there is virtually no consequence of the heterogeneity.

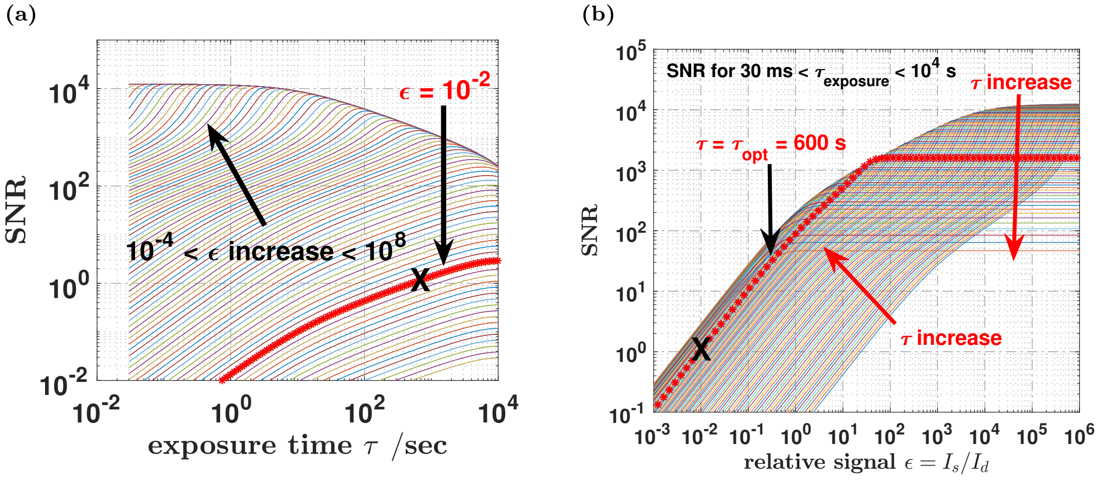

Supplementary figure 4 – **SNR as a function of** τ **and** ϵ. (**a**) shows the SNR as a function of the exposure time τ for a discrete set of values of $10^{-4} < \epsilon < 10^{8}$. (**b**) shows the SNR as a function of the relative intensity of the signal flux $\epsilon = I_s/I_d$, for a ladder of exposure times $30\,\text{ms} < \tau < 10^4$ s. The black cross on both plots show the optimal exposure time τ_{opt} that corresponds to maximal detectivity. The thick red line on (**b**) shows $\text{SNR}_{\tau_{opt}}(\epsilon)$, and indicates the function $\text{SNR}_{\epsilon=10^{-2}}(\tau)$ on (**a**).

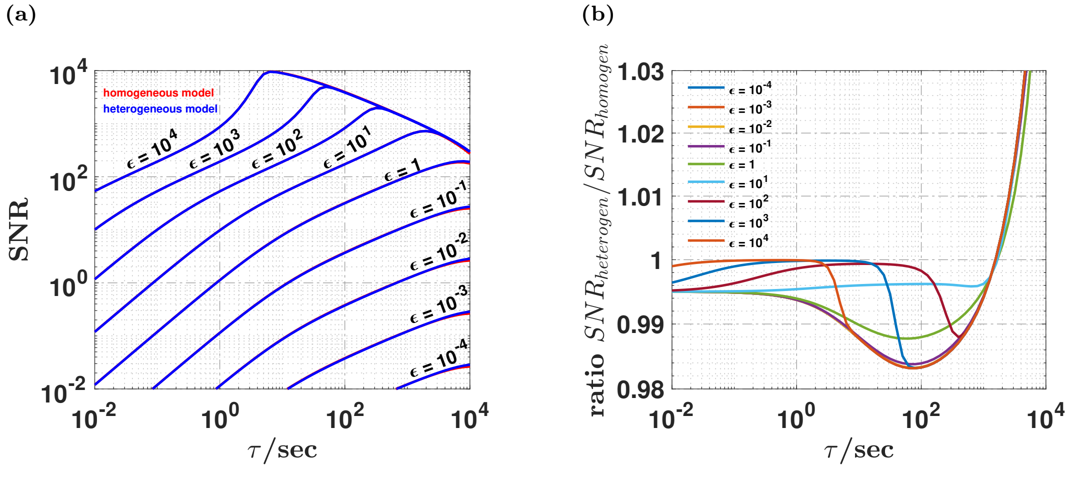

Supplementary figure 5 – **Effect of pixel heterogeneity on the SNR.** We show the results of computing the SNR using the full knowledge (see main figure 1) of the individual pixel characteristics (heterogeneous model), or considering the average characteristics (homogeneous model). The SNR is shown as a function of the exposure time τ, for different values of the signal rate $I_s = \epsilon I_d$ (**b**). (**b**) shows the ratio between the heterogeneous and homogeneous model SNR.

Supplementary note 4 :
Detector instability and excess noise over long time-series.

In this annex, we report on the instability of the detector in complete darkness (see Methods section). Long time-series were acquired under tight temperature control ($\sigma_T \leq 0.02\,°\mathrm{C}$), with different exposure times τ. Each acquisition essentially provides the total count $N1_\tau(t)$ as a random process. To assess the stability of the camera response, we tested the stationarity of that random process. To this end, the statistics of $N1_\tau(t)$ was assessed locally from a sample of k successive values, leading to an estimate of the local mean $< N1_\tau >_{\{k\}} (t)$ and of the local standard error of the mean (SEM) estimated using $k^{-0.5}\sigma^{(k)}_{N1_\tau}(t)$, where $\sigma^{(k)}_{N1_\tau}(t)$ is the experimental dispersion over k adjacent samples.

The non-stationarity and the excess noise at a given time-scale $k\tau$ can be observed by comparing how much the local mean fluctuates compared to the local SEM. Experimental plots shown below do indicate that the detector is not stationary. This observation led us to optimize the sensitivity by a "real-time" noise substraction strategy based on substracting from $N1_\tau(t)$ the noise assessed in with a shutter with $N1_\tau(t \pm \tau)$.

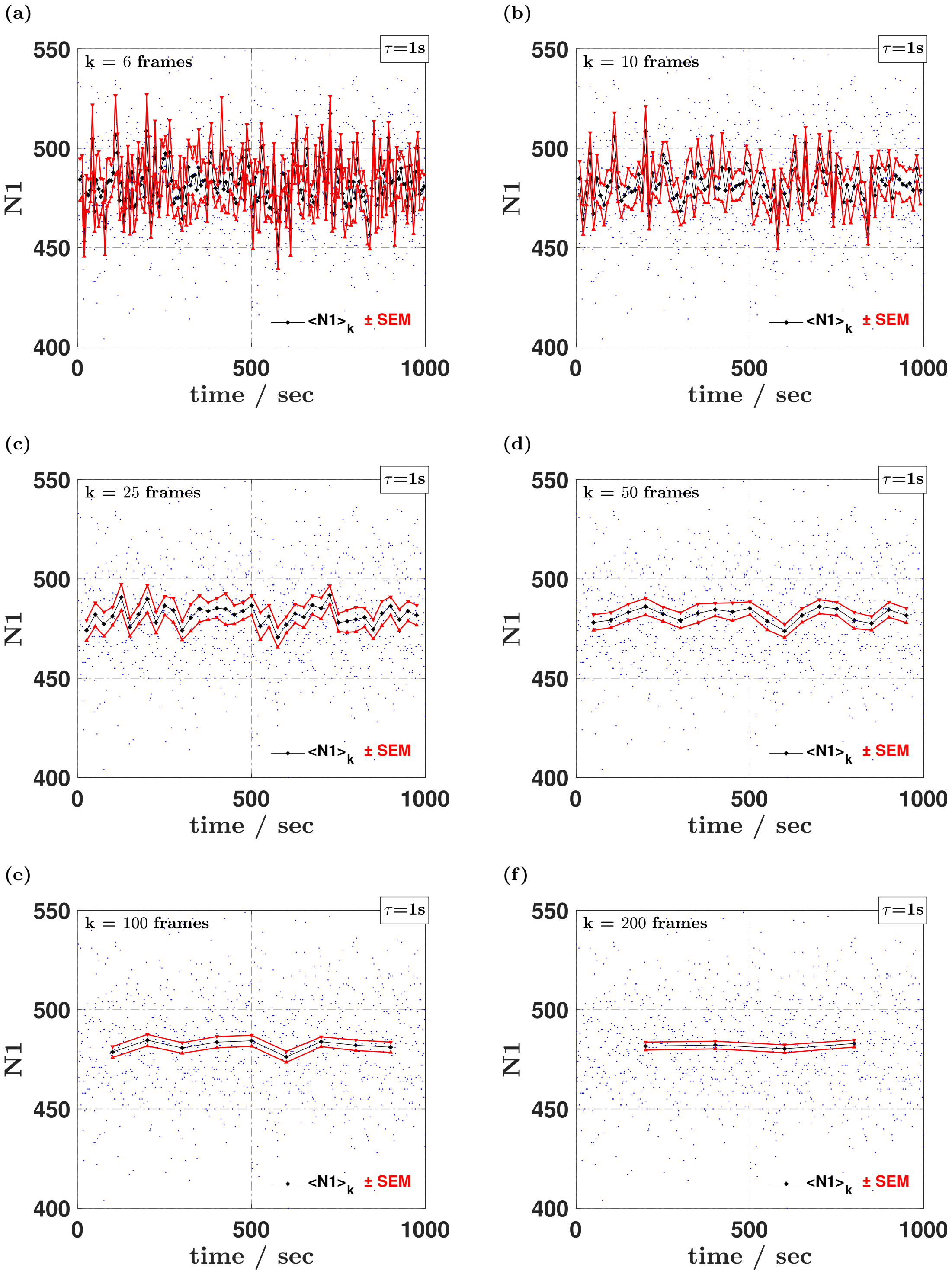

Supplementary figure 6 – **Assessment of detector instabilities -** $\tau = 1\,\mathrm{s}$. The camera was exposed for $\tau = 1\,\mathrm{s}$, and a dataset was acquired from the total count $N1$ measured over 10^3 frames (blue dots). The same dataset was then used to compute a local average $< N1_\tau >_{\{k\}} (t)$ over a moving window of k adjacent frames, together with the standard error of the mean (SEM) $k^{-0.5}\sigma^{(k)}_{N1_\tau}(t)$. The plot show the local statistics, with the average (blue line) with ± 1 SEM (red line), for $k = 6, 10, 25, 50, 100, 200$ frames, (a)-(f).

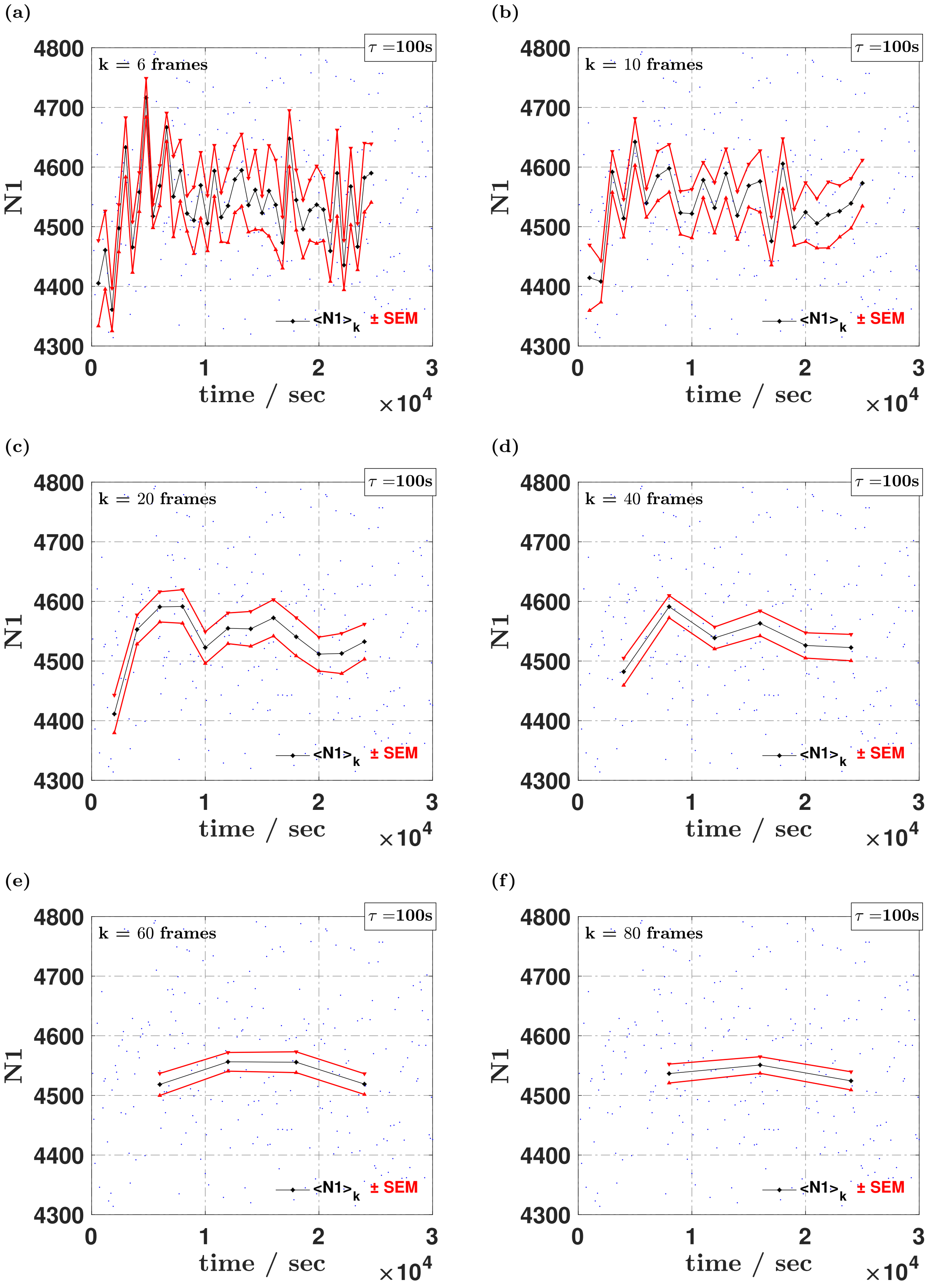

Supplementary figure 7 – Assessment of detector instabilities - $\tau = 100\,$s. The camera was exposed for $\tau = 100\,$s, and a dataset was acquired from the total count $N1$ measured over 300 frames (blue dots). The same dataset was then used to compute a local average $< N1_\tau >_{\{k\}} (t)$ over a moving window of k adjacent frames, together with the standard error of the mean (SEM) $k^{-0.5}\sigma^{(k)}_{N1_\tau}(t)$. The plot show the local statistics, with the average (blue line) with ± 1 SEM (red line), for $k = 6, 10, 20, 40, 60, 80$ frames, (a)-(f).

Supplementary note 5:
Contribution of cosmic rays.

In this note, we provide additional information on how cosmic rays were handled in this work, beyond details given in the Methods section. The observed shift between the cumulative and complementary cumulative distribution functions shown below indicate that cosmic rays contribute in average 4.2x10^{-6} counts/s/pixel in binary counting mode.

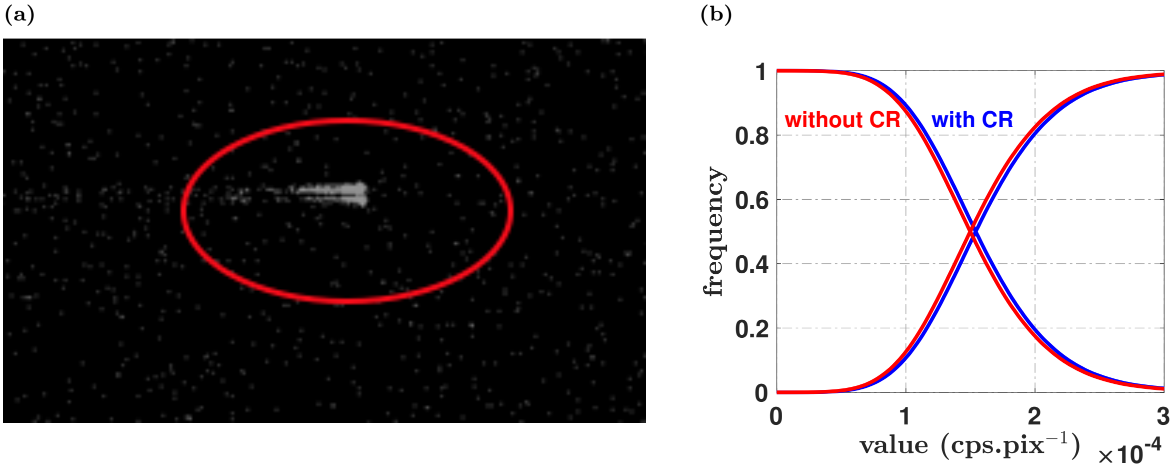

Supplementary figure 8 – **Patterns of cosmic ray (CR) impact, and average contribution** . (a) shows a typical comic ray impact. The total count was assessed over long time series and for a range of exposure times, in the complete darkness. From this data, the dark count rate I_d was assessed for all pixels ij, before and after removing cosmic rays. Two distributions were obtained for $I_{d,ij}$, which are shown (b) in terms of their cumulative and complementary cumulative distribution functions, with (blue line) and without (red lines) CR. The shift which represents the time-averaged contribution of cosmic rays.
